# Assessing the Impact of Spraying an *Enterococcus faecium-*Based Probiotic on Day-Old Broiler Chicks at Hatch on the Incidence of Bacterial Chondronecrosis with Osteomyelitis Lameness Using a *Staphylococcus* Challenge Model

**DOI:** 10.3390/ani14091369

**Published:** 2024-05-02

**Authors:** Anh Dang Trieu Do, Amanda Anthney, Khawla Alharbi, Andi Asnayanti, Antoine Meuter, Adnan Ali Khalaf Alrubaye

**Affiliations:** 1 Cell and Molecular Biology Program, University of Arkansas, Fayetteville, AR 72701, USA; ad086@uark.edu (A.D.T.D.); ka030@uark.edu (K.A.); aasnayan@uark.edu (A.A.); 2 Center of Excellence for Poultry Science, University of Arkansas, Fayetteville, AR 72701, USA; apanthne@uark.edu; 3 National Agency of Drug and Food Control, Jakarta 10520, Indonesia; 4 Animal and Plant Health & Nutrition, Novonesis, 2970 Hørsholm, Denmark; antme@novonesis.com

**Keywords:** broiler, probiotic, lameness, bacterial chondronecrosis with osteomyelitis, *Staphylococcus aureus*

## Abstract

**Simple Summary:**

Bacterial chondronecrosis with osteomyelitis-induced lameness presents a substantial challenge within the avian agricultural sector. The etiology involves pathogenic bacteria translocating from a compromised intestinal barrier into the bloodstream, subsequently colonizing microfractures present in the leg bones that are caused by rapid growth rate and heavy bird weight, resulting in damage and lameness. This study aims to assess whether spraying a non-pathogenic strain of *Enterococcus faecium* bacteria at two different concentrations on day-of-hatch chicks is effective in reducing lameness in broilers using a *Staphylococcus* challenge model. Results indicate that dosing day-old chicks with an effective probiotic reduced lameness incidence in a dose-dependent manner. Findings from this study contribute to the overall understanding of efficient and sustainable broiler production as a high-quality and affordable source of animal protein, while improving bird health and welfare at the same time.

**Abstract:**

Bacterial chondronecrosis with osteomyelitis (BCO) lameness is a bone disease characterized by the translocation of bacteria from the gastrointestinal tract, which colonize microfractures in broiler leg bones caused by rapid animal growth rate and weight gain, resulting in lameness. As such, BCO lameness represents a significant challenge for the poultry industry. This study aims to evaluate the effect of spraying broiler chicks on d0 at hatch with an *Enterococcus faecium* probiotic on the incidence of BCO-induced lameness, utilizing a *Staphylococcus aureus* challenge model. There were four treatments: (1) negative control (no probiotic + no challenge, NC); (2) positive control (no probiotic + challenge, PC); (3) low dosage (4.0 × 10^8^ CFU/chick + challenge, LOW); and (4) high dosage (2.0 × 10^9^ CFU/chick + challenge, HIGH). On d5, groups two through four were challenged with *Staphylococcus aureus* through the drinking water at a concentration of 1.0 × 10^5^ CFU/mL. Cumulative lameness incidence was determined through daily evaluations and necropsies conducted on lame birds starting from d22. Data were subjected to a binomial general regression analysis (significant *p* < 0.05). On d56, the PC group exhibited the highest cumulative lameness incidence (58.0%; *p* < 0.05), followed by LOW (36.0%), HIGH (28.7%), and NC groups (25.3%), respectively. These results suggest early probiotic application at day-of-hatch successfully reduced the incidence of lameness in challenged birds, thus contributing to understanding of efficient and sustainable broiler production.

## 1. Introduction

Domestically and internationally, the poultry industry remains a major player in livestock production in processed volume and affordability for consumers [1]. This holds significant economic importance for an ever-growing world population, whose projected food demand and at-risk population for food hunger are expected to increase by approximately 30% to 62% and −91% to 30% respectively, accounting for changes in global climate [2]. However, with intensive production to meet the increasing demand for animal protein, the industry continues to face multiple production-related diseases that impact animal welfare and productivity, leading to significant economic losses from mortalities and carcass condemnations annually [3,4,5]. Bacterial chondronecrosis with osteomyelitis (BCO) lameness represents one such issue currently facing the industry, affecting approximately 3 to 15% of market-age broilers [6]. Owing to intensive genetic selection for drastic muscling and weight gain rates over the past several decades, modern conventional broiler strains have developed increased susceptibility to this disease due to such gains, which far outpace leg bone development and impose tremendous torque and shear stress on the latter [6,7]. BCO lameness is thought to occur when bacteria translocate from the broiler’s compromised respiratory and gastrointestinal (GI) tracts to the blood and colonize microfractures in the leg bones caused by the rapid growth of the animal [6]. Subsequent worsening infection and eventual necrosis of the bone induce lameness, markedly restricting a bird’s access to feed and water, thus impacting animal welfare and productivity [8]. Several bacterial genera have been known to be associated with this disease, including *Enterococcus* spp. and *Staphylococcus* spp. [9,10]. At present, the diagnostic landscape lacks reliable methods for early detection and effective therapeutics once the clinical presentation of BCO manifests in the late stages of the disease. As such, research into effective preventative measures remains of high relevance and importance for the poultry industry. Over the years, our research group has identified several crucial risk factors to the general understanding of this disease. Following an investigation into supplementation of an organic trace mineral complex including zinc, manganese, and copper in the broiler diet, we determined that such supplementation greatly contributed to intestinal barrier strength via upregulation of tight junction proteins, thus reducing epithelial permeability in the gut and resulting in decreased BCO lameness incidence [11]. In other studies, we also discovered potential inherent predisposing factors of common feed mycotoxins (deoxynivalenol and fumonisin) in BCO lameness pathogenesis [9], as well as the timing of supplement inclusion for optimal lameness reduction outcomes [12]. Finally, in addition to the classical wire-flooring lameness induction model employed in this research [13], we have also developed a novel and effective method of BCO lameness induction that resembles outbreaks in industrial poultry houses by leveraging ventilation airflow that facilitates dissemination of pathogenic organisms to other animals in close vicinity [14], further expanding our repertoire of experimental tools in this study of BCO lameness etiology, pathogenesis, and its mitigation.

Within the last decade, negative consumer perception of animal agriculture and the risk of bacterial antibiotic-resistance has increased. Regulatory restrictions on antibiotic usage in commercial poultry have catalyzed a shift towards the adoption of alternative, natural approaches such as probiotics. These additives, combined with traditional vaccines and good management practices, provide an alternative approach to improving flock health and performance [7,15,16,17,18]. The use of such additives has been postulated to work through several modes of action. For example, exogenous enzymatic additives such as proteases aid in feed digestibility and broiler nutrient absorption [17], thus maximizing feed efficiency. On the other hand, additives belonging to the class of microbiome modulators work by impacting metagenomic functions and metabolic pathways of the microbiome to drive improved health outcomes and performance broilers [16]. With regard to disease prevention and treatment, the mode of action of these additives is thought to vary based on several different mechanisms, including competitive exclusion, improved barrier intestinal barrier function, immunity modulation, and digestion and nutrient absorption [19,20,21]. Similarly, the use of *Enterococcus* as prophylactic probiotics has also been widely studied. As a whole, probiotic *Enterococcus* strains have been known to confer health benefits to the host due to supporting health in the face of adverse conditions. Properties such as competitive exclusion and the production of enterocins—broad-spectrum bacteriocins that have been shown to inhibit growth of pathogenic organisms—may account for some of the beneficial outcomes realized from feeding these probiotic strains [22,23,24,25]. In the poultry industry, *E. faecium* has shown positive outcomes relating to broiler performance, where intestinal health and improved pathogen resistance of young birds have been demonstrated [26,27,28,29]. However, despite encouraging results from utilizing a probiotic in practical evaluations of BCO lameness reduction [30], there remains a severe knowledge gap in the current literature regarding this topic. Additionally, administration routes of probiotic strains in most studies (and in the industry) remain as either a feed component, in drinking water [31], or via in ovo injection [28,32]. Compared to these, direct spraying systems commonly employed in industrial poultry hatcheries and farms may be equally effective while minimizing animal physiological stress compared to methods that require animal handling (such as oculo-oral [33,34]), thereby potentially increasing research translatability when applied to a real-world scenario, using the same treatment of interest. As such, the use of a spraying system in administration of prophylactic probiotics warrants closer examination.

With these considerations, this study aims to assess the effects of administering an effective probiotic in two different concentrations to newly hatched chicks, through a controlled spray mechanism, on cumulative BCO lameness incidence over 56 days of age using a *Staphylococcus aureus* challenge model. We hypothesize that the early exposure and establishment of *E. faecium* in the young chick may help mitigate the negative outcomes resulting from a *S. aureus* challenge, thus reducing subsequent BCO lameness incidence over time. The results of this study may prove valuable to producers seeking to optimize flock health via prophylactic application of probiotics, as well as help ascertain the impact on BCO lameness in broilers. Interestingly, *S. aureus* shares the same prevalence in association with osteomyelitis in humans [35], which also suggests a potential translational model for a disease with high human medical importance.

## 2. Materials and Methods

### 2.1. Environment and Treatment Allocation

This study took place at the University of Arkansas Poultry Environmental Research Laboratory (PERL) from September 13 to November 8, 2023. Cobb 500 male chicks were placed in pens in each of twelve completely isolated environmental chambers, on wood shavings, at a density of 60 chicks per pen on d0 and culled down to 50 birds per pen on d14. The initial pen space dimensions were 1.22 m × 3.51 m (surface area = 4.30 m^2^) from d0–d20 and extended to 2.29 m × 3.66 m on one half (surface area = 6.15 m^2^) from d21 onward to accommodate the birds’ growing sizes. Three pens, each representing a treatment replicate, were allocated to each of the four treatments involved in this study as outlined in Table 1.

As all twelve chambers were completely isolated from one another, pen and block randomization were not conducted. Except for daily health evaluation, feed replenishment, and other emergent issues pertaining to bird caretaking, personnel were encouraged to refrain from entering chambers to minimize disturbance and cross-contamination. Disposable boot covers were changed between movement to each treatment. Each chamber was equipped with temperature regulators to ensure daily bird thermoneutral targets were met, as well as automatic light clocks set at a photoperiod schedule of 23L:1D for the entirety of the study. Each pen was equipped with one water line placed on one end and two hanging feeders on the other to facilitate bird movement. All birds received industry standard formulated starter (crumbles) and finisher (pellets) diets (Table A1) and had access to clean water and feed ad libitum.

### 2.2. Probiotic

The probiotic *E. faecium* strain procured and prepared in the study comes from a commercially available product (GalliPro^®^ Hatch, Novonesis, Hørsholm, Denmark). Per the manufacturer’s specification (2.0 × 10^11^ CFU/g) and recommendation, a low (4.0 × 10^8^ CFU/chick) and high (2.0 × 10^9^ CFU/chick) concentration were calculated and administered via an in-house static spraying system on d0 of age. Boxes of 60 chicks were manually sprayed with multiple passes until exhaustion of a set volume per concentration (LOW = 150 mL/60 chicks; HIGH = 75 mL/60 chicks). Non-toxic blue food dye was added to each prepared stock to aid in visualization of spray dispersion on chicks.

### 2.3. Bacterial Challenge Model

On d5 of the study, except for three negative control (NC) chambers, birds in all remaining treatment chambers received a *S. aureus* challenge in their drinking water via carboys. Glycerol stock *S. aureus* strain used in the study was revived, incubated for 24 hrs with viable CFU concentration determined, and diluted in 20 L of clean water per carboy to a final concentration of 1.0 × 10^5^ CFU/mL. Carboys were vigorously shaken intermittently throughout each day to ensure no settling occurred. All challenged pens received the bacterial water challenge on d5 until exhaustion of the carboys, after which the water source was switched back to clean water.

### 2.4. Lameness Evaluation

**Figure 1:**
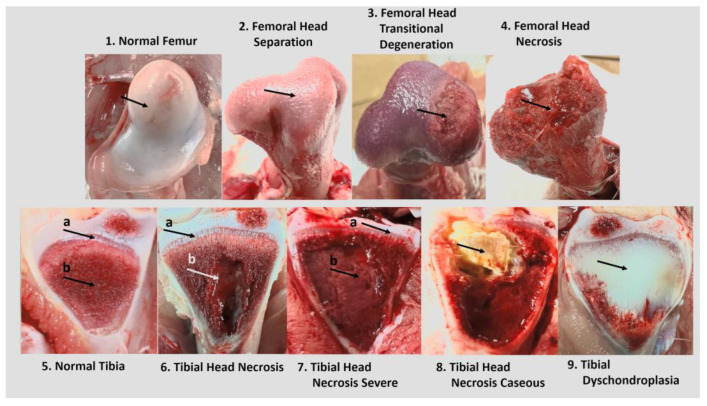
Examples of BCO lesion severity categories for diagnosis. Arrows provided to indicate hallmark characteristics: **1.** Normal proximal femoral head state with intact epiphyseal articular cartilage; **2.** Proximal femoral epiphysis surface separated from cartilage that remains in the acetabulum; **3.** Separated proximal femoral epiphysis with varying degrees of damage (moderate lesion here with fibrinonecrotic exudate); **4.** Extreme damage to fracture of weakened proximal femoral epiphysis and physis upon disarticulation of the femur; **5.** Normal state of proximal tibia with clearly defined physeal growth plate (a) and firm cancellous bone (b); **6.** Necrotic state of the proximal tibia, still with clearly defined physeal growth plate (a) but damaged cancellous bone, replaced with a necrotic void of various sizes (b); **7.** Severe necrotic state of the proximal tibia, with large necrotic void (b) encroaching upon the physeal growth plate (a); **8.** Necrotic state of the proximal tibia with additional caseous exudate, marking bacterial infiltration region; **9.** Proximal tibial head afflicted with tibial dyschondroplasia, marking abnormally large region of cartilage instead of cancellous bone.

Starting from d22 of the study, daily clinical lameness in each pen was evaluated by gently encouraging the birds to walk brief distances. Birds that were reluctant to walk or incapable of walking were diagnosed as clinically lame, euthanized, and necropsied to assess BCO lesions on femoral and tibial heads as per Wideman [6]: N = Femur head and proximal tibia appear entirely normal; FHS = Proximal Femoral Head Separation (epiphyseolysis); FHT = Proximal Femoral Head Transitional degeneration; FHN = Proximal Femoral Head Necrosis; THN = Proximal Tibial Head Necrosis; THNC = Proximal Tibial Head Necrosis Caseous; and THNS = Proximal Tibial Head Necrosis Severe. Other symptoms are TD = Tibial Dyschondroplasia; and KB = Kinky Back (spondylolisthesis). Figure 1 visualizes these lesion progression categories.

### 2.5. Data Analyses

Data were entered and processed using Microsoft^®^ Excel v2403 (Microsoft Corporation, Redmond, WA, USA) from which cumulative lameness incidence over time and lesion categories per treatment (expressed in percentages) were calculated and plotted. Total cumulative lameness data was prepared separately, followed by binomial general regression (or generalized linear model) analysis using JMP^®^ Pro v17.1 (SAS Institute Inc., Cary, NC, USA). All statistical significance was determined at α < 0.05.

## 3. Results

**Figure 2:**
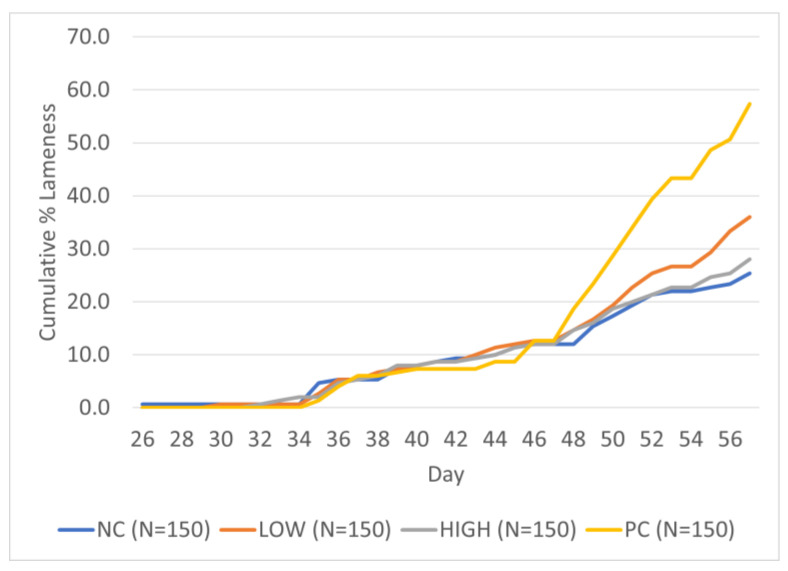
Cumulative percent lameness by treatment groups from d35–57 of the study. Treatments are as follows: NC = Negative Control, PC = Positive Control, LOW = Low, and HIGH = High.

Exhibition of clinical lameness first appeared in the NC group on d26, but not in all treatments until d35 of the study. Cumulative lameness incidence per treatment (in percentage) from d35–57 of the study is presented in Figure 2. 

Cumulative lameness incidence trends between all treatments remained largely similar until d46–47 of the study, following which incidence rate in the positive control (PC) group increased sharply and continued to do so until the end of the study, peaking at 58%, followed by the LOW (L; 36%), HIGH (H; 28.67%), and negative control (NC; 25.33%) groups. Table 2 summarizes the cumulative lameness incidence rate progression for the last four weeks of the study and significant statistical differences between treatments (if any) using binomial generalized regression analysis.

Table 3 outlines binomial logistic regression analysis of total cumulative lameness incidence at the end of the study on d56.

As summarized, PC treatment is significantly different compared to all other treatments involved in the study (*p* < 0.05). Both HIGH and NC groups share a similarly high degree of significance compared to PC (*p* < 1.0 × 10−4), which agrees with their statistical similarity (*p* = 0.52).

**Figure 3:**
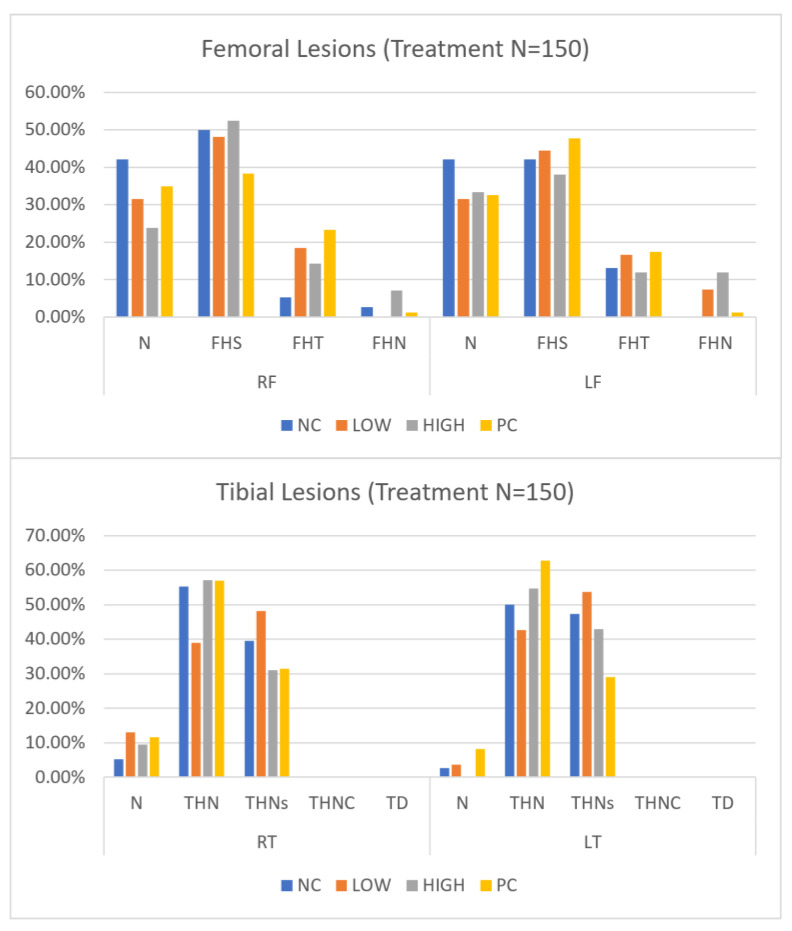
Tibial and femoral lesion severity categories and their incidence rate for clinically lame birds as per Wideman [6]. RT- Right Tibia, LT- Left Tibia, RF- Right Femur, LF- Left Femur, N = Femur head, and proximal tibia appear entirely normal, FHS = Proximal Femoral Head Separation (epiphyseolysis), FHT = Proximal Femoral Head Transitional degeneration, FHN = Proximal Femoral Head Necrosis (bacterial chondronecrosis with osteomyelitis, BCO), THN = Proximal Tibial Head Necrosis, THNC = Proximal Tibial Head Necrosis Caseous, THNS = Proximal Tibial Head Necrosis Severe, TD = Tibial Dyschondroplasia. Treatments are as follows: NC = Negative Control, PC = Positive Control, LOW = Low, and HIGH = High.

An evaluation of BCO lesion distribution among treatments is presented in Figure 3.

Overall, N (normal) and FHS (Femoral Head Separation) are generally the most frequently seen lesion category among femoral lesions, while THN (Tibial Head Necrosis) and THNS (Tibial Head Necrosis Severe) are mostly seen in tibial lesions. Within femoral lesions, the highest incidence rate is recorded with right leg FHS within the HIGH group at 53.49%. Within tibial lesions, THN has the highest rate within the PC group at 63.22%. There are no apparent trends for lesion severity between right and left legs in both femoral and tibial lesions, nor is there a dominating trend between treatments in severity reduction.

## 4. Discussion

As a whole, Enterococci make up a diverse group consisting of mostly commensal and harmless bacteria that can be commonly found inhabiting the GIT of humans and other animal species—poultry included. Application of *Enterococcus*-based probiotics in livestock production has seen increasing adoption, especially as an early intervention strategy. Utilizing these probiotics as a prophylactic supplement has been extensively researched within the poultry industry, demonstrating significant benefits in supporting normal health, performance, and providing protection against potentially harmful bacteria [28,29,32]. Effective probiotics exhibit a wide array of modes of action, including competitive exclusion of potentially harmful bacteria by occupying the same ecological niches and stimulating host immune responses, which enables the bird to mount a better response against challenges [36]. With these mechanisms in mind, Wideman et al. (2012) postulated that inclusion of prophylactic probiotics (including *E. faecium*) in the feed may aid in the reduction of BCO-associated lameness in broilers. The pathogenesis of this is likely based on the “leaky gut” model with translocation of pathogenic bacteria across the gut lining and seeding of the joint infection sites [13]. This wire-floor model provides more physical stress to the growing broilers than what is exerted in a commercial setting. Although this model is useful to evaluate the impacts of experimental products on BCO lameness outcomes in a research setting, it is important to follow up on this research with commercial in-field evaluations. Therefore, a closer examination of this probiotic in a commercial context, such as the day-of-hatch spray application [37] and its impact on BCO lameness is warranted. 

The *Staphylococcus* challenge model used in the study was designed to introduce and establish an abundance of *S. aureus* in the immature chick gut [38], thereby increasing translocation chances of these potentially harmful bacteria across the gut lining following a “leaky gut” event with a bacterium known to be associated with BCO lameness. As such, this model also evaluates if early *E. faecium* supplementation attenuates the effects of oral *S. aureus* challenges on BCO lameness in a dose-dependent manner. The results of this study strongly suggest that that the challenge of *S. aureus* in drinking water on d5 was successful in inducing clinical BCO lameness, indicated by significantly higher cumulative lameness incidence over 57 d of age in challenged versus non-challenged birds (PC vs. NC; 58.00% vs. 25.33%; Table 2). Administration of GalliPro^®^ Hatch on newly hatched chicks on d0 was also found to be effective in reducing cumulative BCO lameness at the end of the study (PC vs. L vs. H; 58.00%, 36.00%, 28.67%, respectively; Table 2). While cumulative lameness incidence rate was still lowest in the NC group, it should be noted that this group received no mechanical or pathogenic challenge over the duration of the experiment beyond an intensive lighting schedule that was applied equally to all treatment groups. Therefore, a lower overall incidence rate in the NC group was expected. Numerical data indicated a higher cumulative incidence of lameness in both probiotic-treated groups; however, the differences were not statistically significant compared to the NC group. This suggests that probiotic treatment demonstrates potential efficacy in mitigating clinical BCO-related lameness. Additionally, a difference of only 3.34% between HIGH and NC groups is almost negligible (*p* = 0.52) and highlights a potential dose-dependent effect at a 5× higher dosage (2.0 × 10^9^ CFU/g) compared to the low dosage. Future research using a similar approach could include measuring levels of enterocins recovered between treatment dosages at different timepoints. This will help better define the probiotic’s mode of action and the impact of dose-response which we hypothesize to correlate to pathogenic microbial composition in the gastrointestinal tract of the animal and subsequent BCO lameness incidence rate.

Progression of BCO lesions commonly observed in various studies at the research site is shown in Figure 1. Generally, FHN (Femoral Head Necrosis) lesions are regarded as the most severe category, indicated by the complete fracturing or necrosis of the proximal femoral head, which is usually coupled with erosion of articular cartilage and loss of smooth joint articulation [39]. Birds afflicted with FHN lesions exhibit reduced mobility—and are often completely unable to move—leading to severe impacts on their health and welfare due to the inability to access feed and water [8]. In contrast to FHN, FHS (Femoral Head Separation) and FHT (Femoral Head Transitional [Degeneration]) lesions present less severe physical damage of the proximal femoral head, indicated by relatively smooth (FHS) to somewhat damaged (FHT) femoral epiphysis. Despite this less severe appearance, birds presenting with these lesions still exhibit an observable negative impact on gait, ranging from reluctance to walk to an unacceptable degree of immobility due to a lack of articular cartilage. Tibial lesions severity progression follows a similar trend to femoral head damage, with an expanding necrotic void encroaching on the growth plate of the proximal tibial head from THN (Tibial Head Necrosis) to THNS (Tibial Head Necrosis Severe). In rare occasions, a caseous exudate may also be present in THNC (Tibial Head Necrosis Caseous) lesions, marking bacterial-associated necrosis [6]. In this study, the reduction in lameness incidence observed in probiotic supplemented groups does not seem to extend to BCO lameness lesion severity, as there were no clear trends between treatments with respect to lesion category or severity, nor were there any regarding leg side (Figure 3). This observation agrees with the current understanding of BCO lameness treatment in that there is a lack of effective therapeutic intervention for BCO lesion severity after onset, which continues to progress until severe lameness is noted. Research to identify preventive approaches for BCO is important because of this lack of therapeutic efficacy and the lack of healing observed in the short grow-out period. In this study, the higher dosage of probiotic inclusion did not adversely affect bird health, observed behavior, or mortality rates. The remaining birds evaluated on d56 of the study showed neither significant differences in body weight nor mortality count between treatment groups (Table A1), indicating normal growth in birds not exhibiting signs of clinical lameness. 

While the results show significant promise of the prophylactic administration of the *E. faecium* in clinical BCO lameness reduction, further investigation is warranted to accurately characterize the influence of this probiotic on the gut microbiota. In humans, the gut microbiota has long been regarded as an important site of high biological relevance, often having lasting influence on the functionality of various systems, including the immune [40] as well as central nervous and enteric nervous systems [41]. In broilers, the gut microbiota are well characterized within the ceca and have been directly linked to animal health and performance [42]. Recently, it has been discovered in humans that surgical site infections (SSI) are closely connected to a patient’s immediate preoperative microbiome [43]. Despite the inherent differences across species, there are grounds to consider the characterization of broiler gut microbial communities and their impacts on BCO lameness etiology and pathogenesis—particularly concerning the concept of a “leaky gut” and its association with infection of leg bone microfractures in a similar fashion to SSIs. Shifts in the microbial populations may also correlate with changes in the intestinal morphology that have been previously documented with the use of GalliPro^®^ Hatch [28]. Finally, while the use of the isolated chambers in this study allowed for evaluation of BCO lameness incidence with the confounding environmental grid factor eliminated, it may be also more commercially relevant to utilize a different induction model in future research, such as the hybrid aerosol model [14] meant to simulate the conditions of large-scale poultry production. Such models leverage horizontal transmission of particulates via ventilation airflow, which closely resembles outbreaks often seen in industrial broiler housing, thus increasing the relevance of future research findings.

## 5. Conclusions

This study was conducted to assess the impact of spraying an *Enterococcus faecium-*based probiotic strain on day-old broiler chicks at hatch on reducing clinical BCO lameness using a *Staphylococcus aureus* bacterial challenge model in drinking water. Cumulative lameness incidence at the end of the study was significantly higher in the challenged untreated PC group compared to the other treatments, while cumulative lameness incidence in the LOW and HIGH probiotic-supplemented groups were not significantly different from the negative control (NC), suggesting probiotic supplementation at day of hatch effectively mitigated BCO-associated lameness. Results also showed a possible dose-response effect between supplemented treatments. This study’s findings hold considerable relevance for the poultry industry in reducing incidence of lameness, directly improving animal health and welfare. Further research is warranted to better understand the mode of action of the probiotic attenuating BCO lesions.

## Figures and Tables

**Table 1:** Detailed study treatment descriptions.

Treatments	Group Descriptions	Animals/Pens
NC	Negative Control	150 birds/3 pens
PC	Positive Control (No spray vaccination + *S. aureus* challenge on d5)	150 birds/3 pens
LOW	Probiotic Concentration #1 (0.25 mL/chick spray vaccination on d0 + *S. aureus* challenge on d5)	150 birds/3 pens
HIGH	Probiotic Concentration #2 (1.25 mL/chick spray vaccination on d0 + *S. aureus* challenge on d5)	150 birds/3 pens

**Table 2:** Cumulative lameness progression per week for last 4 weeks of the study (in %).

Day	NC	PC	LOW	HIGH
35	4.67 ^a^	1.33 ^a^	2.67 ^a^	2.00 ^a^
42	9.33 ^a^	7.33 ^a^	8.67 ^a^	9.33 ^a^
49	15.33 ^a^	24.00 ^a^	16.67 ^a^	16.67 ^a^
56	25.33 ^a^	58.00 ^b^	36.00 ^a^	28.67 ^a^

Superscripts with different letters per timepoint indicate significant statistical differences at α < 0.05.

**Table 3:** Binomial logistic regression of cumulative lameness incidence between treatments at d57.

*p*-Value	PC	LOW	HIGH
NC	<1.0 × 10^−4^ *	0.05	0.52
PC		2.0 × 10^−4^ *	<1.0 × 10^−4^ *
LOW			0.18

Asterisks (*) indicate statistical significance.

## Data Availability

Datasets used or analyzed in the study are available from the corresponding author upon request (A.A.K.A.).

## Appendix A

**Table A1:** Proximate analysis of broiler diets in the study.

Ingredient	Starter	Finisher
Ash, %	5.15	4.76
Dry Mater, %	89.6	89.4
Protein, %	22.5	20.5
Crude Fat, %	4.83	1.35
Al, ppm	80.4	51.5
Ca, ppm	8530	7075
Cu, ppm	114	130
Fe, ppm	452	216
K, ppm	9199	6869
Mg, ppm	1606	1353
Mn, ppm	142	111
Na, ppm	1107	1216
P, ppm	6775	6118
S, ppm	2422	2175
Zn, ppm	142	128

**Table A2:** Average d56 sample bird weights in all treatments (in kg) and total mortality throughout entire study.

Treatment	Number of Birds	Average Weight (±SEM, in kg)	Mortality (Birds)
NC	6	10.76 ± 0.39	0
PC	6	10.86 ± 0.39	1
LOW	6	11.50 ± 0.39	3
HIGH	6	11.74 ± 0.39	1
